# Impact of a Clinical Text–Based Fall Prediction Model on Preventing Extended Hospital Stays for Elderly Inpatients: Model Development and Performance Evaluation

**DOI:** 10.2196/37913

**Published:** 2022-07-27

**Authors:** Yoshimasa Kawazoe, Kiminori Shimamoto, Daisaku Shibata, Emiko Shinohara, Hideaki Kawaguchi, Tomotaka Yamamoto

**Affiliations:** 1 Artificial Intelligence in Healthcare Graduate School of Medicine The University of Tokyo Tokyo Japan; 2 Department of Biomedical Informatics Graduate School of Medicine The University of Tokyo Tokyo Japan; 3 Quantum Computing Center Keio University Tokyo Japan; 4 Department of Performance Monitoring and Risk Management The University of Tokyo Hospital Tokyo Japan

**Keywords:** accidental falls, accident prevention, inpatients, machine learning, natural language processing, propensity score, hospital, elderly, prediction model, patient, risk assessment

## Abstract

**Background:**

Falls may cause elderly people to be bedridden, requiring professional intervention; thus, fall prevention is crucial. The use of electronic health records (EHRs) is expected to provide highly accurate risk assessment and length-of-stay data related to falls, which may be used to estimate the costs and benefits of prevention. However, no studies to date have investigated the extent to which hospital stays could be shortened through fall avoidance resulting from the use of prediction tools.

**Objective:**

We first estimated the extended length of hospital stay caused by falls among elderly inpatients. Next, we developed a model that predicts falls using clinical text as input and evaluated its accuracy. Finally, we estimated the potentially shortened hospital stay that would be made possible by appropriate interventions based on the prediction model.

**Methods:**

Patients aged 65 years or older were selected as subjects, and the EHRs of 1728 falls and 70,586 nonfalls were subjected to analysis. The extended-stay lengths were estimated using propensity score matching of 49 associated variables. Bidirectional encoder representations from transformers and bidirectional long short-term memory methods were used to predict falls from clinical text. The estimated length of stay and the outputs of the prediction model were used to determine stay reductions.

**Results:**

The extended length of hospital stay due to falls was estimated to be 17.8 days (95% CI 16.6-19.0), which dropped to 8.6 days when there were unobserved covariates at an odds ratio of 2.0. The accuracy of the prediction model was as follows: area under the receiver operating characteristic curve, 0.851; F-value, 0.165; recall, 0.737; precision, 0.093; and specificity, 0.839. When assuming interventions with 25% or 100% effectiveness against cases where the model predicted a fall, the stay reduction was estimated at 0.022 and 0.099 days/day, respectively.

**Conclusions:**

The accuracy of the prediction model using clinical text is considered to be higher than the prediction accuracy of conventional assessments. However, our model’s precision remained low at 9.3%. This may be due, in part, to the inclusion of cases in which falls did not occur because of preventative interventions during hospitalization. Nonetheless, it is estimated that interventions for cases when falls were predicted will reduce medical costs by 886 Yen/day (~US $6.50/day) of intervention, even if the preventative effect is 25%. Limitations include the fact that these results cannot be extrapolated to short- or long-term hospitalization cases, and that this was a single-center study.

## Introduction

Falls in older adults represent a serious social issue, as they can cause grave injuries that may result in the victim becoming bedridden and in need of professional care. These risks also exist within medical institutions, where falls among elderly patients considerably contribute toward extended hospital stays, increased costs, and decreased quality of life. The incidence of falls in Japanese hospitals is reported to be 4.40/1000 patient days, and the incidence of falls accompanying disabilities is reported to be 0.29/1000 days [1], which is comparable to the respective values of 3.56/1000 and 0.93/1000 patient days reported in the United States [2].

Risk factors of falls include intrinsic variables such as muscle weakness, history of falls, gait deficit, balance deficit, utilization of assistive devices, visual deficit, arthritis, impaired activities of daily living, depression, and cognitive impairment. Extrinsic risk factors include specific medications, polypharmacy, dark lighting, loose carpets, and a lack of bathroom safety devices [3]. Risk assessment tools are often used by medical institutions to assess the susceptibility to falling based on these risk factors. Morita et al [4] investigated the predictive performance of risk factors using a multivariate logistic regression model on 19 fall-related explanatory variables: (1) age of 70 years or older, (2) previous history of falls, (3) decreased lower-limb muscle strength, (4) use of a cane or walker, (5) wobbling, (6) disturbed behaviors, (7) strong independence, (8) decreased comprehension, (9) overestimation of self, (10) need for someone else to stand by during excretion, (11) need for assistance during excretion, (12) nocturia, (13) narcotics, (14) antidepressants, (15) laxatives, (16) sleep stabilizers, (17) antihypertensive agents, (18) clinical department or room transfers, and (19) oxygen inhalation drip. The results showed that the prediction accuracy reflected an area under the receiver operating characteristic curve (AUC) value of 0.822, a recall of 74.5%, and a specificity of 79.6%. Tools for assessing fall risk factors are commonplace, such as the renowned Morse Fall Scale [5], St. Thomas Risk Assessment Tool [6], Resident Assessment Instrument [7], and Hendrich Fall Risk Model [8]. Their use requires manual responses by health care professionals. Hence, the tendency is for the number of actions to be small, which improves clinician interpretability but may negatively affect the results. Furthermore, there remain significant differences in the input terms applied by medical professionals to electronic health records (EHRs). However, there are expectations that computers will be able to help predict falls with high accuracy and thus improve patient safety.

Among EHR types, clinician-input text data (ie, clinical text) contain information relating to falls, including patient condition. Previous research has applied natural language processing (NLP) techniques to EHR text to classify entries related to falls and to predict whether patients would fall during hospitalization. Toyabe [9] investigated the frequency of true fall event entries from progress notes, discharge summaries, image orders, and incident reports via text mining using dependency parsing. Bjarnadottir et al [10] reported that information on true fall events was most frequently recorded in progress notes (100%), incident reports (65.0%), and image orders (12.5%). They further analyzed intensive care unit nursing records from the Medical Information Mart for Intensive Care database, finding meaningful information related to the risk and prevention of falls [10]. Nakatani et al [11] extracted the nursing records of 335 fallen and 408 unfallen individuals from the EHR system of an acute care hospital, and reported the accuracy of fall prediction by morphological analysis and machine learning methods. The average AUC value from five independent experiments was 0.834 (SD 0.005), and the prediction model contained many words closely related to known risk factors [11]. These studies showed that entries related to patient falls can be extracted from EHRs using NLP, but only with a certain level of accuracy. Nevertheless, fall probability can be predicted during hospitalization, and the results suggest that it may be a useful risk management tool.

To the best of our knowledge, no studies have investigated the extent to which hospital stays could be shortened through fall avoidance resulting from the use of prediction tools. If the extended hospital stay by a fall can be quantitatively classified, then the costs of developing predictive accuracy and preventative measures can be estimated based on the performance of these aspects. Therefore, in this study, the subject demographic was narrowed down to elderly inpatients, and we estimated the extended length of hospital stay caused by falls using the propensity score matching method. In the United States, it has been reported that patients injured by falls during hospitalization had an average stay extension of 6-12 days, incurring additional hospitalization costs of US $13,316 [2,12-14]. However, differences in medical systems and patient demographics compared with those in Japan prohibit the generalization of these figures. Thus, we conducted this investigation anew for Japan. Specifically, we compared the length of hospital stay in fallen and unfallen groups with adjustments made for patient demographics, which were obtained by propensity score matching using 49 covariates that are considered to influence both falls and length of hospital stay to ultimately estimate the average treatment effect on treatment (ATET). Additionally, the effect of unobserved covariates on ATET was investigated using sensitivity analysis. Next, we used clinical entries made at the time of hospitalization of an elderly inpatient with annotations of the presence or absence of a fall to create a data set. The proposed method was built upon bidirectional encoder representations from transformers (BERT) [15], a general-purpose NLP model. Predictions were made by inputting the clinical text up to the second day of hospitalization and setting the objective of prediction as whether the patient would fall within the next 30 days of hospitalization. Finally, the results were used as a basis to estimate the shortened length of hospital stay and reduced medical costs as a result of fall prevention measures. We then investigated the potential costs incurred in implementing the model and the associated precautionary measures.

## Methods

### Data Set

**Figure 1:**
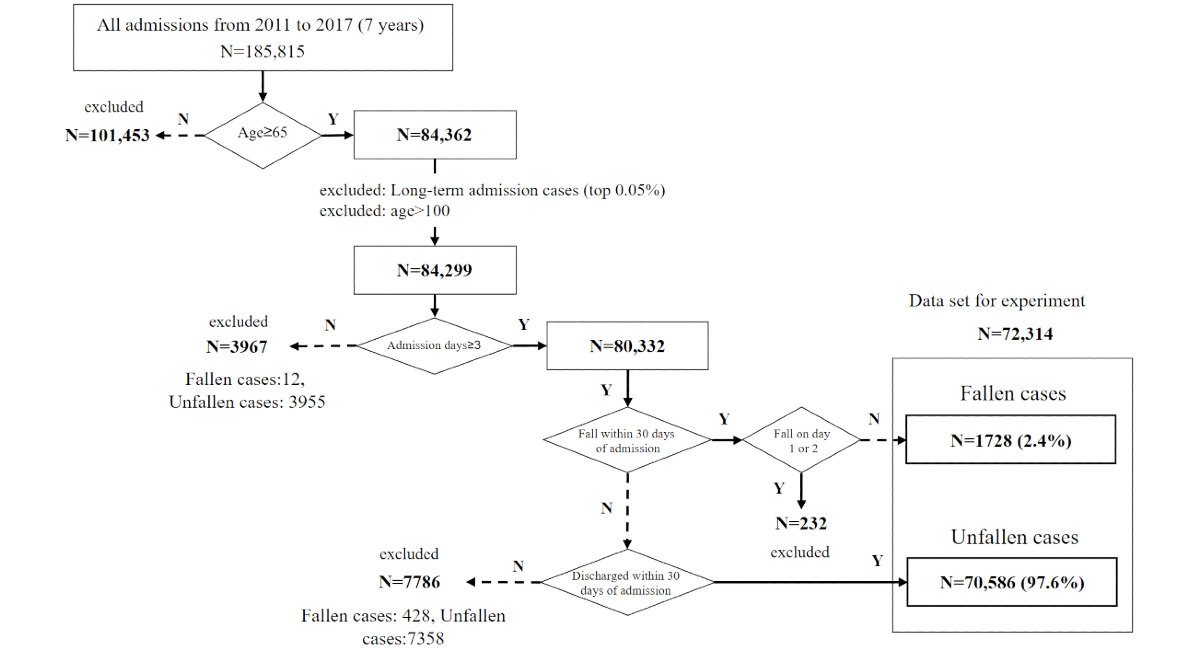
Flowchart of data collection and selection procedure.

Among all hospitalizations that overlapped in the 7-year period from January 1, 2011, to December 31, 2017, patients aged 65 years or older at the time of hospitalization were included. However, those with a hospitalization period that was extensive (top 0.05% number of days) and those aged 100 years or older were excluded as outliers. As a result, a total of 84,299 hospitalizations were obtained from the EHR system of the University of Tokyo Hospital. Results of comparing these hospitalizations with the occurrence of falls that were reported in incident reports indicated that 2402 falls were reported and 82,089 were not. In the second half of this study, we used clinical text from the first 2 days of hospitalization to predict the occurrence of falls from subsequent days. However, it was considered that predicting future falls from 2 days’ worth of clinical text would be difficult. Therefore, the prediction period was limited to the period from day 3 to day 30 of hospitalization, during which falls resulted in the hospitalization being classified as “fallen hospitalization” and the nonoccurrence of a fall resulted in the hospitalization being classified as “unfallen hospitalization.” Experimental subjects included 72,314 cases (1727 fallen and 70,586 unfallen) after excluding those among the previously mentioned 84,299 that did not meet all criteria. Figure 1 shows the extraction flow of the experiment subjects.

### Ethics Approval

All experiments and data collection were approved by the institutional review board at the University of Tokyo Hospital (approval number 201919NI). All experiments described below were carried out under relevant ethical guidelines and regulations.

### Variables

#### Occurrence of Falls

We used falls that were reported in incident reports, which have a high degree of completeness, as such reporting is mandatory. These reports distinguish between falls during walking and falls from bed, including mild and severe classifications. However, these cases were not classified separately in this study.

#### Risk Factors for Falls

Factors other than falls influence the length of hospital stay; thus, determining the extended length of stay caused by falls requires the elimination of covariates that affect both falls and the length of hospital stay. A total of 49 covariates were identified by propensity score matching to adjust their effects on diagnosis procedure combinations (DPCs), incident report data sets, blood test results, and prescription drugs.

DPCs contain information entered by medical staff for all inpatients regarding diagnostic procedures. We used several factors influencing falls and length of stay, including age, gender, consciousness disorder at admission, emergency transport at admission, dementia at admission, purpose for chemotherapy at admission, and the disease that triggered hospitalization. The latter was coded using the 10th revision of the International Statistical Classification of Diseases and Related Health Problems, and 17 types of dummy variables were developed based on the major classification code (A to U). All variables, apart from age, were treated as binary variables.

Incident reports comprise systematic reviews showing that past falls are high predictors of subsequent falls [16-18]. Previous history includes cases of hospitalization where falls were reported in the respective incident report.

Blood test results were used to determine the presence or absence of anemia and poor nutritional status, which are known risk factors that affect falls. Seven variables were adapted as test results reflecting these risks, including decreased hemoglobin, decreased protein/albumin, increased urea nitrogen (suggesting chronic kidney disease), increased liver enzymes, decreased blood glucose, abnormal electrolytes, and elevated C-reactive protein. Each threshold value was set as a binary variable. Table A1 in Multimedia Appendix 1 provides the thresholds for each variable.

Prescription drugs in this case include hypnotics and antipsychotics, which have been identified as contributors to falls [3]. Binary variables were set for these drugs using the criteria of the drug corresponding to its three-digit drug efficacy classification code from the subcategory “87 drugs and related products” of the Japanese standard product classification. The following 12 drug groups were considered: hypnotics, antiepileptics, nonsteroidal anti-inflammatory drugs (NSAIDs), anti-Parkinson drugs, antipsychiatric drugs, other neuroactive drugs, muscle relaxants, diuretics, antihypertensive drugs, diabetes drugs, narcotics, and laxatives. Furthermore, polypharmacy is known to contribute to falls. This includes cases in which 10 or more of the above-mentioned drugs were prescribed simultaneously.

#### Clinical Notes

Clinical text was used as input to the fall prediction model without distinguishing the type of clinician.

#### Period of Data Extraction

**Figure 2:**
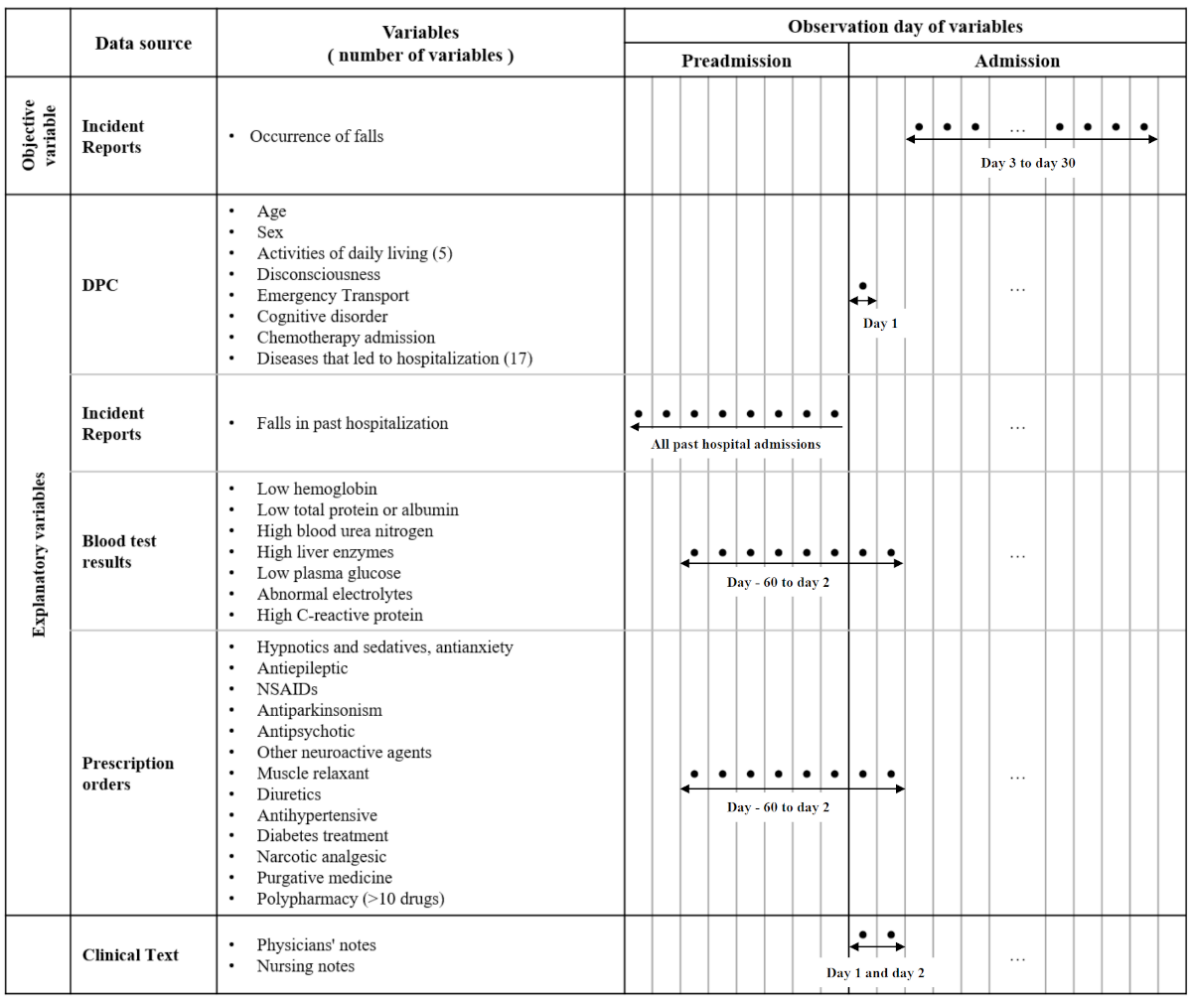
Period and variables of data extraction. DPC: diagnosis procedure combination; NSAID: nonsteroidal anti-inflammatory drug.

It was desirable to obtain the above-mentioned 49 variables from the clinical text entered on the day of hospitalization. However, there was a concern that the number of missing values would increase if the target period for variable extraction was limited to that day. Therefore, variables relating to blood test results and prescribed drugs were taken from the 60 days before hospitalization to the second day of hospitalization. For the clinical text used as input, the subject period included the first and second days. Figure 2 shows the variables used and their target periods.

### Missing Values

Figure 2 shows that there were no missing values found in the DPC data. However, the blood test results and prescription orders showed cases in which these entries did not exist during the target period. These missing values were estimated using the multiple imputation by chained equation (MICE) method 20 times [19].

### ATET Estimation by Propensity Score Matching

The extended length of hospital stay caused by falling was estimated using propensity score matching [20]. Matching unfallen cases with tendencies similar to those of fallen cases and comparing the lengths of hospital stays between the two groups were achieved by repeating this method, resulting in an ATET estimation of the effect of falls on the length of stay. The propensity score was obtained using a multivariate logistic regression model with the 49 explanatory variables and the presence or absence of falls as the objective variable. Some variables had missing values, as described above. Thus, values estimated from 20 MICE calculations were used as inputs to the multivariate logistic regression model. The one-to-one nearest-neighbor matching with replacement method [21] was used to match the fallen and unfallen groups. Here, propensity score matching estimations strongly assumed that the fall allocation depended only on the explanatory variables used; however, not all variables were observed. Therefore, the effects of the unobserved ATET covariates were also investigated using sensitivity analysis to the maximum *P* value and minimum Hodges-Lehmann point estimate [22] according to Rosenbaum’s [23,24] procedure. Here, the null hypothesis is *fall events do not influence the extended length of hospital stay*, and the *P* value is the value of the one-sided Wilcoxon signed-rank sum test.

### NLP Fall Prediction From Clinical Text

Fall prediction learning and evaluation were performed on 71,943 cases, excluding 371 cases with missing clinical text from the 72,314 experimental data, as shown in Figure 1. Cases in which hospitalization occurred between 2011 and 2016 (1500 fallen and 60,060 unfallen) were used as learning data; cases in which the day of hospitalization was in 2017 (228 fallen and 10,158 unfallen) were divided into two groups so that the number of fall cases was even. Subsequently, two-fold cross-validation was performed using alternating models for model selection and evaluation. The AUC, F-value, recall, precision, and specificity were used as evaluation indicators, and the 2-time average value was used for performance evaluation.

**Figure 3:**
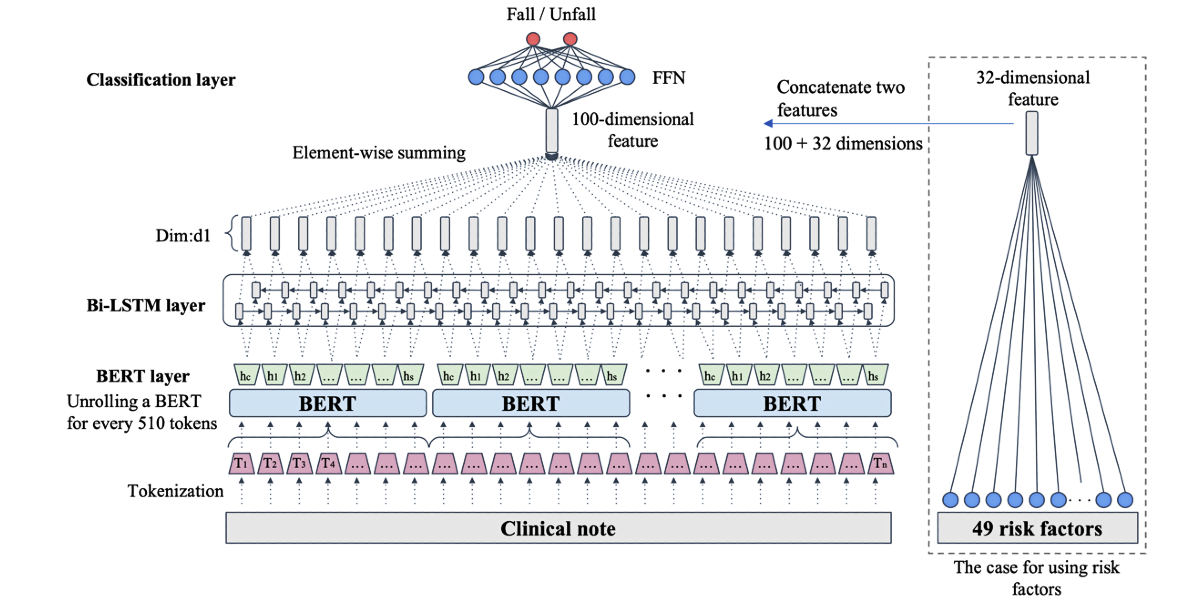
Overview of the bidirectional encoder representations from transformers (BERT) classification model. The input document was divided into 510 tokens; classification [CLS] and separation [SEP] tokens were added at each end, and the input was sequential. All token embeddings output sequentially were used as inputs to the bidirectional long short-term memory (Bi-LSTM) model, and the 50-dimensional vectors in the forward and reverse directions that were output for each were combined to form 100-dimensional vectors. The feature value obtained from the document was set as the sum of each dimension of the multiple 100-dimensional vectors, which were converted linearly and output as binary fallen or unfallen values using a sigmoid function. FFN: feedforward neural network.

We adopted a model that leveraged UTH-BERT [25], which was pretrained on Japanese clinical text using bidirectional long short-term memory (Bi-LSTM) [26] to predict falls. The model-learning process involved dividing clinical text into vocabulary tokens unique to UTH-BERT, and adding the special tokens for classification ([CLS]) and separation ([SEP]) to the beginning and end of sequences. In BERT, a fixed-length sequence of up to 512 tokens is taken as input, and the embeddings of [CLS] and those corresponding to each input token are considered as output. [CLS] embeddings are used as input to the classifier, after which fine tuning is performed [15]. Owing to this limitation, it was proposed to divide the input by 512 so that the tokens could be input sequentially. In this way, [CLS] embeddings could be output sequentially to a classifier (eg, recurrent neural network) that can use the series to classify sentences consisting of longer sequences [27]. However, [CLS] embeddings do not always aggregate the contents of an entire sentence, and the likelihood of reduced performance was a concern [28]. Therefore, we instead adopted a model in which the output of BERT token embedding was input into a single-layer Bi-LSTM so that a 100-dimensional feature value output could be obtained. This was then used to perform the binary classification of fallen and unfallen cases. Furthermore, the structure provided that a 32-dimensional feature value would be obtained by linearly converting the 49 fall-related variables from the clinical text, followed by their binary classification. Figure 3 shows the structure of the BERT+Bi-LSTM network.

The median number of characters in the clinical text of fallen and unfallen cases was 4144 and 2105, respectively, and the amount of text used to describe fallen cases tended to be larger. Additionally, the median number of tokens obtained from tokenizing the UTH-BERT vocabulary was 2531 and 1288, respectively. The sequential input of long sequences to BERT required maintaining an error gradient; thus, GPU memory limitations resulted in the curtailment of the input token (text) length. In this study, we used eight Tesla-V-100 processors with 16 GB GPU memory. However, there was a limit of 13 BERT inputs (6630 tokens; 510 tokens×13). Therefore, text exceeding this limit had to be truncated. There were a total of 444 hospitalization cases that exceeded 6630 tokens, which comprised 0.6% of the entire data set. Ultimately, it was determined that this limitation would not have a large effect on model performance.

### Measures Against Imbalanced Data

Since the number of fallen and unfallen cases was uneven (see Figure 1), to reduce the impact of imbalanced data on learning, the inverse of the class frequency calculated from the training data set was weighted to the loss function. This is a simple heuristic method that is widely adopted in the presence of class imbalance [29].

### Experimental Settings

We evaluated the performance of three prediction models: two-layer multilayer perceptron (inputs=49 fall-related variables), BERT+Bi-LSTM (inputs=only clinical text), and BERT+Bi-LSTM (inputs=clinical text+49 fall-related variables). For all prediction models, output binary values for each fallen and unfallen case were obtained using a sigmoid function to minimize the value with cross-entropy loss. It was determined that the learning stop condition would occur when the AUC value stopped improving for five epochs. Performance differences between the models were then investigated via net reclassification improvement (NRI) [30]. MeCab [31] was used as the morphological analyzer of the clinical text, and Mecab-ipadic-Neologd [32] and the Japanese disease name dictionary [33] were used as analyzer dictionaries. To develop the prediction models, we used Python v.3.8.5 (Python Software Foundation) and the PyTorch v.1.7.1 machine learning framework (Facebook’s Artificial Intelligence Research Lab). All statistical analyses were conducted using the STATA v.16.1 integrated statistical software package.

## Results

### Fall-Related Variables

Table 1 lists the mean value, missing value rate, adjusted odds ratio, and standardized difference of the 49 fall-related variables. The average length of hospital stay was 30.3 days (SD 23.7) for fallen hospitalization and 10.6 days (SD 6.8) for unfallen hospitalization, with the difference being 19.7 days. No missing values were found in the basic patient and disease characteristics. The variable with the most missing values in the blood test results was plasma glucose at a missing rate of 19.7%. The missing value rate of variables related to prescription drugs was 8.3%. The 20-time AUC average was 0.73 (95% CI 0.72-0.74). Variables showing a significant difference at *P*<.05 for basic patient information included age, gender, assistance in bathing and movement in activities of daily living, impaired consciousness at admission, and previous history of falls in past admissions. Similarly, several diseases were significantly correlated with falls in terms of hospitalization triggers: diseases of the blood and blood-forming organs, mental and behavioral disorders, diseases of the eye and adnexa, diseases of the circulatory system, diseases of the digestive system, diseases of the skin and subcutaneous tissue, and diseases of the musculoskeletal system and connective tissue. For blood tests, low hemoglobin, low total protein or albumin, and abnormal electrolytes were significantly correlated with falls. For prescription drugs, NSAIDs, anti-Parkinson drugs, antipsychotics, other neuroactive agents, and diuretics were significantly correlated with falls. Among all fall-related variables, mental and behavioral disorders had the highest odds ratio and diseases of the eye and adnexa had the lowest odds ratio (Table 1).

**Table 1:** Statistics of fall-related variables.

Variables			Fallen cases (n=1728)		Unfallen cases (n=70,586)		Multivariate regression^a^					Standardized difference		
							Adjusted odds ratio (95% CI)		*P* value^b^		Before matching (n=1728 fallen cases, n=70,586 unfallen cases)			After matching (n=1728 fallen cases, n=1728 unfallen cases)
Hospital days, mean (SD)			30.3 (23.7)		10.6 (6.8)		N/A^c^		N/A		N/A			N/A
**Demographics**														
	Age (years), mean (SD)	76.5 (6.8)		74.3 (SD 6.4)		1.03 (1.02-1.03)^d^		<.001		0.33			–0.04	
	Sex (male 0, female 1), positive rate (%)	40.6		43.8		0.71 (0.63-0.80)		<.001		–0.06			0.01	
	ADL^e^ Eats, positive rate (%)	9.2		2.4		1.08 (0.83-1.40)		.57		0.29			0.04	
	ADL Bathe, positive rate (%)	19.2		5.5		1.37 (1.06-1.77)		.02		0.43			0.04	
	ADL Dressing^f^, positive rate (%)	15.3		4.4		0.76 (0.57-1.02)		.07		0.37			0.04	
	ADL Transferring^g^, positive rate (%)	26.2		8.6		1.79 (1.48-2.18)		<.001		0.48			0.02	
	ADL Continence^h^, positive rate (%)	13.0		3.5		1.04 (0.80-1.37)		.75		0.35			0.05	
	Unconsciousness (JCS^i^ 0,≠0), positive rate (%)	18.1		5.6		1.70 (1.44-2.00)		<.001		0.39			0.04	
	Emergency transport, positive rate (%)	8.6		3.9		0.96 (0.78-1.17)		.68		0.20			–0.01	
	Cognitive disorder, positive rate (%)	11.1		4.0		1.10 (0.92-1.32)		.28		0.01			0.01	
	Chemotherapy admission, positive rate (%)	11.7		11.4		1.08 (0.91-1.27)		.39		0.27			0.02	
	Past fallen, positive rate (%)	8.1		3.5		1.37 (1.13-1.65)		.001		0.20			0.01	
**Disease**														
	Certain infectious and parasitic diseases (A00-B99), positive rate (%)	8.6		6.8		0.98 (0.82-1.17)		.78		0.07			–0.02	
	Neoplasms (C00-D48), positive rate (%)	40.8		41.1		1.10 (0.97-1.25)		.12		–0.01			–0.01	
	Diseases of the blood and blood-forming organs (D50-D89), positive rate (%)	8.3		6.3		1.28 (1.07-1.53)		.01		0.08			–0.002	
	Endocrine, nutritional, and metabolic diseases (E00-E90), positive rate (%)	23.8		18.5		1.09 (0.96-1.24)		.19		0.13			–0.01	
	Mental and behavioral disorders (F00-F99), positive rate (%)	4.6		1.1		2.09 (1.61-2.71)		<.001		0.21			0.05	
	Diseases of the nervous system (G00-G99), positive rate (%)	8.4		4.7		1.14 (0.95-1.38)		.16		0.16			–0.001	
	Diseases of the eye and adnexa (H00-H59), positive rate (%)	3.8		13.3		0.47 (0.36-0.61)		<.001		–0.35			0.04	
	Diseases of the ear and mastoid process (H60-H95), positive rate (%)	0.3		0.8		0.47 (0.19-1.14)		.10		–0.07			0.01	
	Diseases of the circulatory system (I00-I99), positive rate (%)	33.9		26.1		1.15 (1.02-1.29)		.02		0.17			0.00	
	Diseases of the respiratory system (J00-J99), positive rate (%)	9.5		6.2		1.01 (0.85-1.20)		.91		0.12			–0.01	
	Diseases of the digestive system (K00-K93), positive rate (%)	17.8		16.6		0.77 (0.67-0.87)		<.001		0.03			–0.01	
	Diseases of the skin and subcutaneous tissue (L00-L99), positive rate (%)	3.0		1.6		1.46 (1.09-1.95)		.01		0.09			–0.00	
	Diseases of the musculoskeletal system and connective tissue (M00-M99), positive rate (%)	11.9		8.4		1.22 (1.04-1.43)		.02		0.11			0.00	
	Diseases of the genitourinary system (N00-N99), positive rate (%)	10.0		7.3		0.94 (0.79-1.12)		.50		0.10			–0.003	
	Pregnancy, perinatal period, congenital malformations (O00-Q99), positive rate (%)	0.3		0.4		1.03 (0.42-2.52)		.94		–0.10			0.00	
	Symptoms, signs, and abnormal clinical and laboratory findings (R00-R99), positive rate (%)	5.8		3.3		1.03 (0.83-1.28)		.80		0.12			–0.01	
	Injury, poisoning and certain other consequences of external causes (S00-T98), positive rate (%)	5.1		3.1		1.11 (0.88-1.40)		.38		0.10			0.00	
**Blood tests**														
	Low hemoglobin (3.9% missing data), positive rate (%)	71.8		57.5		1.34 (1.19-1.53)		<.001		0.24			–0.04	
	Low total protein or albumin (5.0% missing data), positive rate (%)	48.7		33.8		1.20 (1.08-1.34)		.001		0.32			–0.04	
	High blood urea nitrogen (4.4% missing data), positive rate (%)	3.4		1.6		1.20 (0.90-1.61)		.22		0.12			–0.004	
	High liver enzymes (AST^j^, ALT^k^; 4.0% missing data), positive rate (%)	6.0		3.6		1.22 (0.98-1.52)		.07		0.12			–0.01	
	Low plasma glucose (19.7% missing data), positive rate (%)	2.5		1.7		1.14 (0.80-1.62)		.48		0.05			–0.01	
	Abnormal electrolytes (Na, K, Cl; 12.1% missing data), positive rate (%)	35.1		21.6		1.40 (1.26-1.57)		<.001		0.32			–0.02	
	High C-reactive protein (6.8% missing data), positive rate (%)	10.9		5.0		1.12 (0.94-1.34)		.21		0.22			–0.005	
**Prescription**														
	Hypnotics and sedatives, antianxietics	37.4		30.7		1.09 (0.97-1.22)		.13		0.13			–0.001	
	Antiepileptic	4.4		1.8		1.30 (1.00-1.69)		.05		0.16			0.03	
	NSAIDs^l^	43.5		32.6		1.21 (1.08-1.36)		.001		0.22			–0.03	
	Antiparkinsonism	3.2		1.0		1.61 (1.18-2.21)		.003		0.16			0.02	
	Antipsychotic	21.4		9.6		1.44 (1.25-1.66)		<.001		0.33			0.02	
	Other neuroactive agents	13.8		6.6		1.19 (1.01-1.39)		.03		0.23			0.01	
	Muscle relaxant	0.3		0.1		1.70 (0.60-4.85)		.32		0.03			0.01	
	Diuretic	23.4		13.7		1.33 (1.16-1.53)		<.001		0.24			–0.003	
	Antihypertensive	31.4		25.9		0.88 (0.77-1.00)		.05		0.11			–0.01	
	Diabetes treatment	15.9		12.7		1.08 (0.92-1.26)		.48		0.09			–0.01	
	Narcotic analgesic	3.3		1.4		1.11 (0.81-1.51)		.52		0.12			–0.001	
	Purgative medicine	38.3		32.6		1.09 (0.97-1.22)		.15		0.11			0.00	
	Polypharmacy (>10 drugs)	48.7		35.8		1.02 (0.89-1.17)		.77		0.26			–0.004	

^a^Multivariate logistic regression on the results of missing value estimation by the multiple imputation method.

^b^Based on the two-tailed Z-test for a coefficient of zero.

^c^N/A: not applicable.

^d^The odds ratio for age was calculated by univariate logistic regression with the age range from 65 to 99 years equally transformed from 0.0 to 1.0.

^e^ADL: activities of daily living.

^f^Assistance is required for dressing or personal maintenance.

^g^Assistance is required for walking, going up and down stairs, getting into/out of bed or chair, or going to the toilet.

^h^Assistance is required for either defecation or urination.

^i^JCS: Japan Coma Scale, which has been widely used to assess patients’ consciousness level in Japan.

^j^AST: aspartate aminotransferase.

^k^ALT: alanine aminotransferase.

^l^NSAID: nonsteroidal anti-inflammatory drug.

### Impact of Falls on Hospital Stay

**Figure 4:**
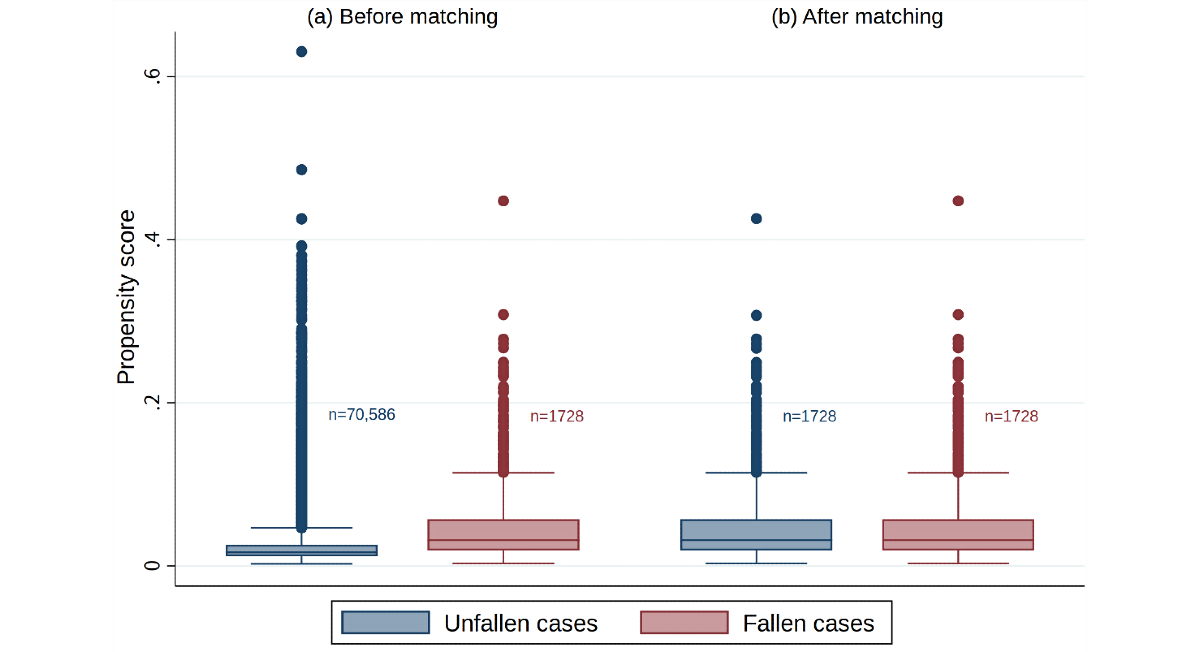
Box-and-whisker plots of the propensity scores (a) before matching and (b) after matching. Boxes show lower and upper IQR, and whiskers show the highest and lowest values, excluding outliers (>1.5 times IQR; rounds). Propensity score matching was performed using one-to-one nearest-neighbor matching with the replacement method on fallen cases.

The AUC of the logistic regression model for which the propensity score was calculated was 0.73. Figure 4 shows the distribution of propensity scores before and after matching. The upper IQR was distributed at a low range of less than 0.2 both before and after matching. The results of matching the fallen and unfallen cases showed a sample size of 1728 for each, and the distribution of propensity scores in each group was similar. Furthermore, as shown in Table 1, the standardized differences [20] for all variables after matching were less than 0.1, and the differences between groups became sufficiently small for all variables [20]. The average length of hospital stays in the unfallen group, in which propensity score matching was performed, was 12.5 days (SD 7.0) and the ATET was 17.8 days (95% CI 16.6-19.0). Based on these results, it was estimated that the average length of hospital stay was extended by 17.8 to 30.3 days from 12.5 days, which was the estimated average length of hospital stay if the fallen cases had not fallen as a result of an elderly inpatient falling.

Table 2 summarizes the results of the Rosenbaum sensitivity analysis for the estimated ATET according to the upper limit of the extent of influence of the unobserved variables on the fall propensity score (Γ), which corresponds to the upper limit when the odds of allocation to a fallen case of the matched pair fluctuate in the range of (1/Γ,Γ) due to the unobserved variables. The maximum *P* value and minimum Hodges–Lehmann point estimate [22] reflect the maximum value of the null hypothesis’ significance level and the minimum ATET value for each Γ value, respectively. Here, the null hypothesis is fall events do not influence the extended length of hospital stay, and the *P* value is the value of the one-sided Wilcoxon signed-rank sum test.

As shown in Table 2, when Γ was 7.5, the lower limit of the estimated value of ATET was 0.8 days, and the null hypothesis could not be rejected at the significance level of .05. By contrast, when Γ<7.5, a significant causal effect was observed. The bias of Γ=7.5 was huge [23], and the robustness of the hypothesis that falls cause an increased length of stay is demonstrated. Furthermore, as shown in Table 1, the highest odds ratio among the 49 covariates was 2.09 for mental and behavioral disorders. However, even with Γ=2.0, which assumes the presence of unobserved factors having the same degree of influence as the above variables, it was estimated that the length of hospital stays of fallen inpatients was extended by at least 8.6 days.

**Table 2:** Sensitivity analysis for *P* value and Rosenbaum bounds estimates (average values calculated over 20 imputed data sets) to unobserved biases.

Γ^a^	Maximum *P* value^b^	Minimum Hodges–Lehmann point estimate (days)
1.0	<.001	14.1
2.0	<.001	8.6
3.0	<.001	6.0
4.0	<.001	4.1
5.0	<.001	2.9
6.0	<.001	2.0
7.0	0.01	1.1
7.5	.05	0.8
8.0	.16	0.5

^a^Γ: odds of differential assignment due to unobserved factors.

^b^The *P* value is based on a one-tailed Wilcoxon signed-rank test for the null hypothesis of no extension of hospital stay caused by falls.

### Performance of Fall Prediction Models

Table 3 summarizes the evaluation results of the machine learning models. Model 1, a multilayer perceptron with only the 49 fall-related factors as input, had the lowest AUC at 0.735. Model 2, the BERT+Bi-LSTM with only the clinical text as input, had the highest AUC at 0.851. Model 3, the BERT+Bi-LSTM using the clinical text and 49 fall-related factors as input, had an AUC of 0.850.

Tables A2 and A3 in Multimedia Appendix 1 list the NRIs for the reclassifications conducted to investigate the performance differences between models. Table A2 shows the result of comparing models 1 and 3; the NRIs of the fallen and unfallen cases were 0.123 (*P*<.001) and 0.068 (*P*<.001), respectively, and the integrated NRI was 0.191 (*P*<.001). This result showed that the performance of Model 3 was significantly improved over that of Model 1, suggesting that using clinical text improved predictive performance. Table A3 shows the result of comparing models 2 and 3, and the integrated NRI of the fallen and unfallen cases was –0.015 (*P*=.48), with no significant differences observed. This result indicates that there were no significant improvements to the performance of Model 3 over Model 2. Thus, adding the 49 fall-related factors to the clinical text did not improve the predictive performance.

**Table 3:** Performance comparison of machine learning models with input data categories.

Model	Input data			Evaluation accuracy^a^				
	49 fall-related factors	Clinical text	AUC^b^		F1^c^	Sensitivity	Specificity	Precision
Model 1: MLP^d^	✓		0.735		0.090	0.662	0.708	0.048
Model 2: BERT^e^+Bi-LSTM^f^		✓	0.851		0.165	0.737	0.839	0.093
Model 3: BERT+Bi-LSTM	✓	✓	0.850		0.138	0.794	0.776	0.076

^a^The accuracies are the average values of two cross-validation tests based on the cutoff determined by the Youden index.

^b^AUC: area under the receiver operating characteristic curve.

^c^F1 is the harmonic mean of precision and recall.

^d^MLP: multilayer perceptron.

^e^BERT: bidirectional encoder representations from transformers.

^f^Bi-LSTM: bidirectional long short-term memory.

### Impact of Prediction-Based Interventions

**Figure 5:**
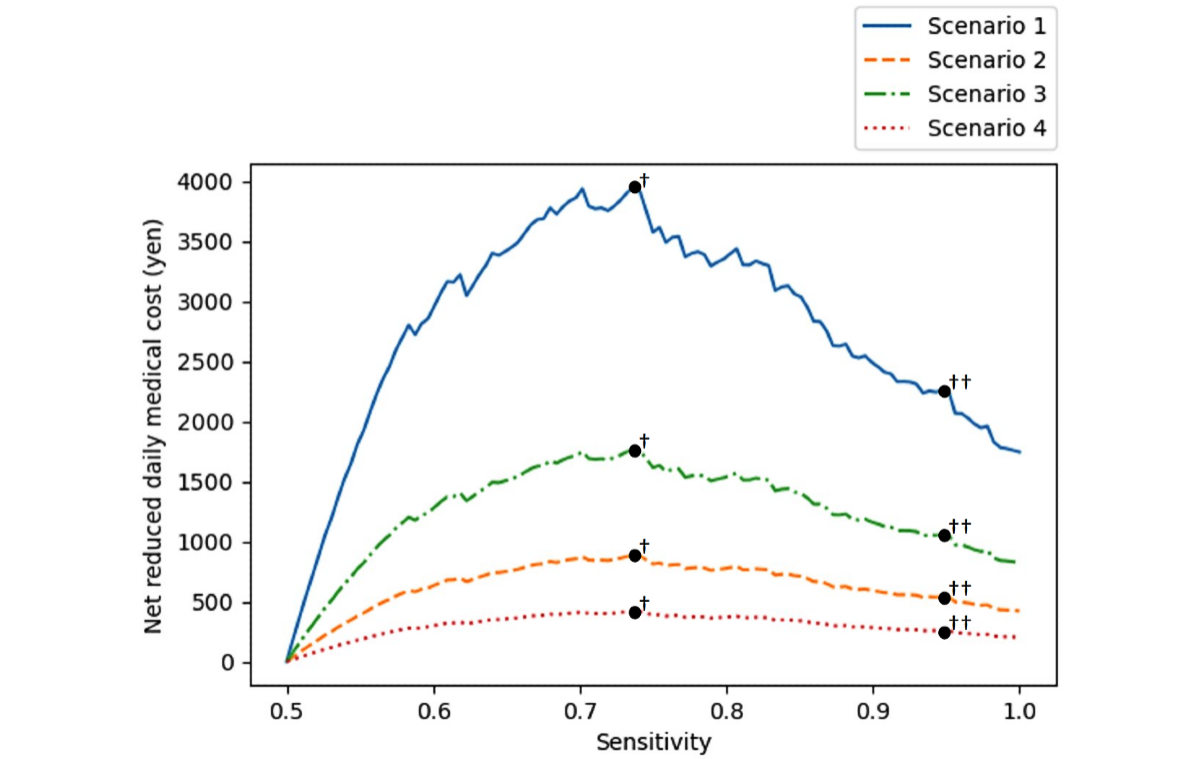
Estimated net reduced daily medical costs by interventions based on Model 2 sensitivity. The maximum points in Scenarios 1-4 are indicated by a circle with † and their values are 3951, 886, 1768, and 420 (Yen; US $1=~136 Yen), respectively. These are taken with a sensitivity of 0.737; the sensitivity is the same as determined using the Youden index. The points with 0.95 sensitivity in Scenarios 1-4 are indicated by a circle with ††, and their values are 2249, 538, 1054, and 258 (Yen), respectively.

Table 4 shows a cross-table summary of the evaluation results of two Model 2 cross-validations based on the cutoff determined by the Youden index. It can be assumed from these results that some positive interventions were conducted on the 1806 hospitalization cases predicted to result in a fall and that some falls were completely prevented across 19,463 days (168 hospitalizations×12.5 days=average days of unfallen cases matched to fallen cases; 1638 hospitalizations×10.6 days=average days of unfallen cases). As a result, the hospitalized stay was shortened by a total of 2990 days (168 hospitalizations×17.8 days=ATET) among cases that were otherwise destined to experience a fall. This corresponds to 0.154 days per day of interventions (2990/19,463 days). Of the 8580 cases that were predicted to be unfallen, 60 cases actually experienced a fall (ie, false negatives). This indicates that 1068 (60 hospitalizations×17.8 days=ATET) shortened hospitalization stays were lost. Thus, the net reduced length of hospital stay was 1922 days (2990–1068 days). This corresponds to 0.099 days per day of interventions (1922/19,463 days). The average daily hospitalization cost in Japan is approximately 40,000 Yen (US $1=~136 Yen) [34]. Thus, the net reduced daily medical costs by active intervention were estimated to have been approximately 3950 Yen (1922 days×40,000 Yen per day/19,463 days) per day of interventions. This interpretation assumes that the preventive effect of aggressive intervention was 100%. However, Table 5 presents estimates when the presumed effect was adjusted to 25.5% and the ATET was set to 8.6 days. While the results up to this point were based on fixed cut-off values determined by the Youden index, Figure 5 shows how the net reduced daily medical costs for scenarios 1-4 in Table 5 change when the cutoff is changed. In Figure 5, the horizontal axis shows the sensitivity to changing the cutoff of Model 2 in the range of sensitivity≥0.5; the vertical axis shows the net reduced daily medical cost. For example, if the sensitivity is set to 0.95, the net reduced daily medical costs are 2249, 538, 1054, and 258 Yen, respectively.

**Table 4:** Cross-table summary of the results of the two Model 3 cross-validations. The cutoff was determined using the Youden index.

Predictions	Fallen cases	Unfallen cases	Sum
Predicted fallen cases	168	1638	1806
Predicted unfallen cases	60	8520	8580
Sum	228	10,158	10,386

**Table 5:** Estimated hospital days reduced by interventions based on Model 2 predictions (sensitivity 73.7%, precision 9.3%).

Scenario	ATET^a^ (days)	Fall prevention rate (%)	Reduced length of hospital stay (number of days per day of interventions)	Hospital stays that could not be reduced (number of days per day of interventions)	Net reduced length of hospital stay (number of days per day of interventions)	Net reduced daily medical costs (Yen per day)^b^
Scenario 1	17.8	100	0.154	0.055	0.099	3950
Scenario 2	17.8	25	0.035	0.012	0.022	886
Scenario 3	8.6	100	0.069	0.025	0.044	1769
Scenario 4	8.6	25	0.017	0.006	0.011	420

^a^ATET: average treatment effect on treatment.

^b^Medical costs were estimated at 40,000 Yen per day (US $1=~136 Yen).

## Discussion

### Principal Results

In this study, we verified the performance of a fall prediction model using clinical EHR text pertaining to elderly patients, and we estimated the reduction in medical costs incurred if fall prevention interventions had been successfully conducted according to the prediction results.

### Extended Hospital Stays Due to Fall

The extended length of hospital stay due to falls (ATET) was estimated at 17.8 days. This value was 1.9 days shorter than the simple difference (19.7 days) between the average days of hospitalized stay between fallen and unfallen groups. This is the result of a positive correlation between fall susceptibility and length of stay, with the exclusion of confounding background factors between groups. Falls include incidental falls, which intuitively lead to 17.8 days of extended stay. In these examples, the analysis subject was aged 65 years or older and was hospitalized for 3 days or more. It is also common for severe falls to result in extended hospitalized stays of 1 month or longer. Thus, it is further intuitive that this may be the effect of averaging incidents and accidents. Meanwhile, this ATET was obtained from 49 variables automatically extracted from the EHR system; thus, there may have been unobserved covariates. The verification of the *P* value of causal effect and robustness of the ATET by Rosenbaum sensitivity analysis showed that the causal effect of falls extending the length of hospitalized stay was significant at a level of *P*<.05, even when assuming unobserved covariates with large odds ratios such as Γ=7. As reported in previous studies [2,12-14], this supports the finding that falls extend the length of hospital stay. Moreover, when assuming a more realistic Γ, of the 49 variables shown in Table 1, if there were unobserved covariates with Γ=2.0 corresponding to mental and behavioral disorders (the largest odds ratio), then the extended length of stay caused by falls was estimated to be at least 8.6 days. This value falls within the 6-12 days reported in US studies [2,12-14]. However, comparisons between acute-care hospitalized stays in 2019 [35] showed an average length of hospital stay in the United States of 6.1 days. The average length of hospital stay in Japan was 16.0 days, which is 2.5 times longer. Therefore, it is intuitive that the extended length of hospital stay due to falls will be longer in Japan. Thus, the extension of 8.6 days is thought to be conservative.

### Fall Prediction Model Performance

The accuracy of the proposed prediction model was investigated by comparing the prediction accuracy of the 19-item multivariate logistic regression model (AUC 0.82), including nurse observations, performed in a previous study [4]. The AUC of Model 1 (multilayer perceptron), which used only the 49 fall-related variables, was 0.735. This was lower than the AUC of 0.82 obtained in the previous study, which used items obtained only by nurse observations as explanatory variables for the multivariate logistic regression models, including decreased lower-limb muscle strength, use of a cane or walker, wobbling, disturbing behaviors, strong independence, and decreased comprehension. These variables are known to affect prediction accuracy. The fact that such items were not included in the 49 variables in this study is clearly the reason for the relatively low accuracy of Model 1. However, the AUC of Model 3, in which clinical text was added, was 0.850. Additionally, because this study evaluated generalization performance using past data for learning and future data for evaluation, this value is intuitively higher than the AUC of 0.82. As described below, clinical nurse risk assessments of falls and fall prevention interventions may have improved model performance.

The AUC of Model 3, which used clinical text, was more than 0.1 higher than that of Model 1, which did not use clinical text. A two-sided Z-test of the NRI between models showed that Model 3 was significantly more accurate. It is therefore rational to conclude that the prediction accuracy of a model that uses clinical text is high because, at the time of hospitalization, the nurse observes the patient, conducts a risk assessment, and records the evaluation results as necessary. Therefore, clinical text contained more information related to fall risk than the 49 fall-related variables, which likely contributed to the improvement in prediction accuracy. Meanwhile, no significant difference was found between the prediction accuracy of Models 2 and 3, suggesting that the clinical text also contained information corresponding to the 49 variables at the time of hospitalization.

It has often been reported that BERT exhibits high performance, even with clinical text [36-39]. This is also true for this study, in which a model combining BERT and Bi-LSTM using clinical text recorded in daily practice allowed for fall prediction with an accuracy equal to or higher than that of conventional risk assessment tools. Although not limited to BERT, prediction models that use neural networks also show high performance. However, they lack a means of explaining the prediction, as opposed to linear and tree models. Application of explanatory techniques such as SHapley Additive exPlanations [40] would lead to remarkable explanatory findings related to falls. Hence, this is a future study direction.

Regarding model precision, Model 2 had the best precision of 9.3%, which was higher than the value of 6.9% obtained in previous research [4]. However, this shows that many false positives were likely present. Predicting a patient’s future is an inherently difficult task; however, the data set used in this study involved fall prevention measures based on risk-assessment results. Thus, it is thought that there were likely some cases in which falls were prevented when the risk was high. Fall prevention measures include a mat-type buzzer installed inside the bed and a mechanism that sounds like a buzzer when the patient leaves the bed. A limitation of this study is that the data set did not contain information about this and other prevention measures. Hence, future studies should not rule these out.

### Impact of Fall Prevention Interventions Based on the Prediction Model

Table 5 shows four scenarios in which the length of hospital stay was shortened when assuming that active fall prevention was conducted for all cases in which Model 2 predicted falls. The net reduced length of hospital stay per day of interventions was 0.099 days/day when the preventive effect was set to 100% (Scenario 1) and 0.022 days/day when the effect was set to 25% (Scenario 2). Additionally, when assuming the presence of unobserved covariates with odds ratios equivalent to 2.0 times, the shortened number of days was 0.044 days/day (Scenario 3) and 0.011 days/day (Scenario 4). The results showed that in cases where medical expenses per day of hospitalization were 40,000 Yen/day, the break-even costs of 3950-420 Yen/day in Scenarios 1-4 were found based on the costs of introducing the prediction model and fall prevention measures. Figure 5, which shows the net reduced daily medical costs when the cutoff changed, reveals that the break-even cost of Scenarios 1-4 was 2249-258 Yen/day when the sensitivity was set to 0.95. Although not shown in Figure 5, as an extreme cut-off setting, the net reduced daily medical costs of applying fall prevention interventions to all cases without using the prediction model were 1469, 357, 696, and 172 Yen in Scenarios 1-4, respectively. There are sensitivity points at which the net reduced daily medical cost is higher using our prediction model than without the prediction model in all scenarios, which shows the advantage of using our prediction model over not using the prediction model. Medical expenses vary depending on the size of the hospital; thus, the break-even point is higher in larger hospitals. Hence, the incentive for prediction should be high. These results reflect the costs of introducing preventive measures in addition to those already taken. Thus, more effective preventive measures are needed. An ideal solution would be to include methods to further prevent falls by attaching a motion sensor to patients when a fall is predicted and using its data to predict near-future behaviors. These technologies are expected to be available in the near future. Furthermore, higher prediction performance and improved fall prevention intervention will further reduce hospitalized stays and medical costs.

### Limitations and Future Work

One limitation of this study pertains to extant preventive measures that may have negated true positives. Another limitation pertains to the results of this study not being applicable to patients with short-term (1-2 days) or long-term (31 days or more) hospital stays. In this study, 232 cases of falls that occurred during the first or second day of hospitalization were excluded. However, these constituted 11.8% of the 1960 total cases (Figure 1). Additionally, although our data set was relatively large, it was limited in that it was obtained from a single facility; thus, it is not generalizable to all of Japan. Future studies should obtain more robust data using multicenter information and analyze the prediction results using techniques that visualize the basis of prediction.

### Conclusions

In this study, it was estimated that the general length of hospital stay in Japan was extended by 17.8 days due to falls among elderly inpatients. The predictive performance of the proposed model, which predicts falls up to the 30th day of hospitalization using clinical text from the second day of hospitalization, showed an AUC of 0.85. Thus, it was suggested that this may be more accurate than traditional risk assessment tools. However, its precision was still low, at 9.3%. A possible reason for this discrepancy may be the inclusion of cases where falls did not occur because of successful fall prevention interventions during hospitalization, which were not accounted for. Fall prevention interventions for cases predicted by this model were shown to reduce medical costs by up to 886 Yen per day, even if the preventive effect was as low as 25%. Limitations include the fact that short- and long-term patients were not included, and only a single-center demographic was applied.

## Appendix

Multimedia Appendix 1 Tables A1-A3.

## Abbreviations

ATET: average treatment effect on treatment
AUC: area under the receiver operating characteristic curve
BERT: bidirectional encoder representations from transformers
Bi-LSTM: bidirectional long short-term memory
CLS: classification
DPC: diagnosis procedure combination
EHR: electronic health record
MICE: multiple imputation by chained equation
NLP: natural language processing
NRI: net reclassification improvement
NSAID: nonsteroidal anti-inflammatory drug
SEP: separation
