# Supplementary material for: Impact of a Clinical Text–Based Fall Prediction Model on Preventing Extended Hospital Stays for Elderly Inpatients: Model Development and Performance Evaluation
Source: JMIR Med Inform. 2022 Jul 27;10(7):e37913. doi: 10.2196/37913 (PMC9377461; doi:10.2196/37913)
Supplement: Multimedia Appendix 1 [file medinform_v10i7e37913_app1.docx]

**Multimedia Appendix 1. Tables A1-A3.**

Table A1. Thresholds of blood test results.

| Variables | Thresholds | | | Coding |
| --- | --- | --- | --- | --- |
|  | Test | Upper limit (unit) | Lower limit (unit) |  |
| Low hemoglobin (Hb) | Hb | Male: 11.6 (g/dL)  Female: 13.7 (g/dL) | - | Set to one if the value is less than the lower limit, other to zero. |
| Low total protein or albumin (TP, Alb) | TP | 6.6 (g/dL) | - | Set to one if the value is less than the lower limit of total protein or albumin, other to zero. |
|  | Alb | 4.1 (g/dL) | - |  |
| High blood urea nitrogen (BUN) | BUN | - | 20.0 (mg/dL) | Set to one if the value is three times or more than the upper limit, other to zero. |
| High Liver enzymes (AST, ALT) | AST | - | 30.0 (IU/L) | Set to one if the value is three times or more than the upper limit, other to zero. |
|  | ALP | - | 322.0 (IU/L) |  |
|  | GGTP | - | Male: 64.0 (IU/L)  Female: 32.0 (IU/L) |  |
| Low plasma glucose (PG) | PG | 73.0 (mg/dL) |  | Set to one if the value is less than the lower limit, other to zero. |
| Abnormal electrolytes (Na, K, CL) | Na | 138.0 (mEq/L) | 145.0 (mEq/L) | Set to one if any one of the values is less than the lower limit or greater than the upper limit, other to zero. |
|  | K | 3.6 (mEq/L) | 4.8 (mEq/L) |  |
|  | CL | 101.0 (mEq/L) | 108 (mEq/L) |  |
| High C-reactive protein (CRP) | CPR | - | 0.3 (mg/dL) | Set to one if the value is 30 times or more than the upper limit, other to zero. |

Table A2. Reclassification comparing two prediction models. Model 1: A two-layer perceptron with 49 factors as input; Model 3: UTH-BERT+Bi-LSTM with clinical text and 49 factors as input. The cutoff was determined using the Youden index.

|  | Prediction results  of Model 1 | | Prediction results  of Model 3 | | NRI  (95% CI)  P-value | |
| --- | --- | --- | --- | --- | --- | --- |
|  |  |  | Fallen  cases | Unfallen cases |  |  |
| Fallen cases  (n = 228) | Fallen cases | 151 | 133 | 18 | 0.123  (0.056 - 0.190)  p < 0.01* | 0.191  (0.123 - 0.258)  p < 0.01* |
|  | Unfallen cases | 77 | 46 | 31 |  |  |
| Unfallen cases  (n = 10,158) | Fallen cases | 2,966 | 1,422 | 1,544 | 0.068  (0.058 - 0.077)  p <0.01* |  |
|  | Unfallen cases | 7,192 | 856 | 6,336 |  |  |

NRI is the net reclassification improvement. P-value is based on two-tailed Z-tests for an expected NRI of zero. * Indicates a statistically significant difference (i.e., p < 0.05). CI stands for confidence interval.

Table A3. Reclassification comparing two prediction models. Model 2: UTH-BERT+Bi-LSTM with clinical text as input; Model 3: UTH-BERT+Bi-LSTM with clinical text and 49 factors as input. The cutoff was determined using the Youden index.

|  | Prediction results  of Model 2 | | Prediction results  of Model 3 | | NRI  (95% CI)  P-value | |
| --- | --- | --- | --- | --- | --- | --- |
|  |  |  | Fallen  cases | Unfallen  cases |  |  |
| Fallen cases  (n = 228) | Fallen cases | 168 | 162 | 6 | 0.048  (0.008 - 0.089)  p = 0.02* | -0.015  (-0.056 - 0.027)  p = 0.48 |
|  | Unfallen cases | 60 | 17 | 43 |  |  |
| Unfallen cases  (n = 10,158) | Fallen cases | 1,638 | 1,343 | 295 | -0.063  (-0.070 - -0.056)  p < 0.01* |  |
|  | Unfallen cases | 8,520 | 935 | 7,585 |  |  |

NRI is the net reclassification improvement. P-value is based on two-tailed Z-tests for an expected NRI of zero. * Indicates a statistically significant difference (i.e., p < 0.05). CI stands for confidence interval.
